# MicroRNA-877-5p promotes osteoblast differentiation by targeting EIF4G2 expression

**DOI:** 10.1186/s13018-023-04396-y

**Published:** 2024-02-12

**Authors:** YingChao Shen, Yang Zhang, Qiang Wang, Bo Jiang, XiaoWei Jiang, Bin Luo

**Affiliations:** 1 Department of Orthopaedics, Changshu Hospital Affiliated to Nanjing University of Chinese Medicine, No. 6 Huanghe Road, ChangShu City, 215500 China; 2 School of Biology and Food Engineering, Changshu Institute of Technology, Changshu City, 215500 Jiangsu China; 3 Department of Hand and Foot Surgery, The Second Affiliated Hospital of Soochow University, Jiangsu Province, No. 1055 Sanxiang Road, Suzhou City, 215004 China

**Keywords:** MC3T3-E1, miR-877-5p, EIF4G2, Osteoblast differentiation

## Abstract

Stimulating bone formation potentially suggests therapeutics for orthopedic diseases including osteoporosis and osteoarthritis. Osteoblasts are key to bone remodeling because they act as the only bone-forming cells. miR-877-5p has a chondrocyte-improving function in osteoarthritis, but its effect on osteoblast differentiation is unknown. Here, miR-877-5p-mediated osteoblast differentiation was studied. Real-time reverse transcriptase-polymerase chain reaction was performed to measure miR-877-5p expression during the osteogenic differentiation of MC3T3-E1 cells. Osteoblast markers, including alkaline phosphatase (ALP), collagen type I a1 chain, and osteopontin, were measured and detected by alizarin red staining and ALP staining. Potential targets of miR-877-5p were predicted from three different algorithms: starBase (http://starbase.sysu.edu.cn/), PITA (http://genie.weizmann.ac.il/pubs/mir07/mir07_data.html), and miRanda (http://www.microrna.org/microrna/home.do). It was further verified by dual luciferase reporter gene assay. The experimental results found that miR-877-5p was upregulated during the osteogenic differentiation of MC3T3-E1 cells. Overexpression of miR-877-5p promoted osteogenic differentiation, which was characterized by increased cell mineralization, ALP activity, and osteogenesis-related gene expression. Knockdown of miR-877-5p produced the opposite result. Dual luciferase reporter gene assay showed that miR-877-5p directly targeted eukaryotic translation initiation factor 4γ2 (EIF4G2). Overexpression of EIF4G2 inhibited osteogenic differentiation and reversed the promoting effect of overexpression of miR-135-5p on osteogenic differentiation. These results indicate that miR-877-5p might have a therapeutic application related to its promotion of bone formation through targeting EIF4G2.

**Supplementary Information:**

The online version contains supplementary material available at 10.1186/s13018-023-04396-y.

## Introduction

Osteoporosis is a globally prevalent bone condition connected with bone resorption and loss of the bone microstructure, which can lead to bone fragility and increased bone fracture [1]. One of the key methods to manage the imbalance in bone mass is to stimulate osteoblast formation [2]. Osteoblasts, as the main cell type for bone formation, are the key to the metabolic homeostasis, growth, and injury repair of bone tissue [3–5], and some protein markers, such as alkaline phosphatase (ALP), collagen type I a1 chain (COL1A1), and osteopontin (OPN), are believed to have value as bone development biomarkers [6]. Therefore, investigation into the regulatory mechanisms behind osteoblast differentiation will aid in the diagnosis or treatment of osteoporosis.

Osteoblast differentiation is closely regulated by a variety of factors [7–9], among which miRNAs have recently received attention. miRNAs exert their regulatory functions by regulating their target genes at the post-transcriptional level, thereby participating in various biological processes [10–14]. miR-25 [15], miR-33-5p [16], miR-221, and miR-15b [17] have been revealed to act on corresponding targets, thus affecting the balance between bone formation and resorption, and therein regulating osteoblast differentiation. miR-877-5p is frequently downregulated in various cancers [18–20], and particularly, miR-877-5p has an alleviating impact on osteoarthritis chondrocytes [21]. In addition, miR-877-3p promotes osteoblast differentiation of MC3T3-E1 cells by targeting Smad7 [22]. However, the role of miR-877-3p in osteoblast differentiation has not been studied.

Translation of most mRNAs is regulated at the initiation level, a process that requires a protein complex called EIF4F [23]. Eukaryotic translation initiation factor 4γ (EIF4G) has two functional homologs in mammals, namely EIF4G1 and EIF4G2 [24]. EIF4G2 has been studied in osteoarthritic cartilage and has an association with chondrocytes in the pathogenesis of osteoarthritis [25]. Potential targets of miR-877-5p were predicted from three different algorithms: starBase (http://starbase.sysu.edu.cn/), PITA (http://genie.weizmann.ac.il/pubs/mir07/mir07_data.html), and miRanda (http://www.microrna.org/microrna/home.do). The results showed that miR-877-5p and EIF4G2 had targeted binding sites. Moreover, the targeting relationship between miR-877-5p and EIF4G2 was further verified by dual luciferase reporter gene assay.

Therefore, this study aimed to investigate the role of miR-877-5p and EIF4G2 in osteoblast differentiation and found that miR-877-5p promoted osteoblast differentiation through targeted regulation of EIF4G2 expression. Our results may be useful in enhancing new bone formation and designing treatments for pathological bone loss.

## Methods

### Cell culture

Mouse osteogenic MC3T3-E1 cells (Cell Bank of Chinese Academy of Sciences) were cultured in Dulbecco's modified Eagle medium containing 10% fetal bovine serum (FBS), 100 U/ml penicillin, and 100 mg/ml streptomycin. Osteoblast differentiation induction medium was prepared by α-minimum essential medium (Hyclone), 10% FBS, 1% penicillin/streptomycin, 50 ug/mL l-ascorbic acid, 10 mM β-glycerophosphate, and 10 nM dexamethasone. At 3, 7, 14, and 21 d after osteogenic induction, miR-877-5p and EIF4G2 levels were checked.

### MC3T3-E1 cell transfection

miR-877-5p mimic (5′-GUAGAGGAGAUGGCGCAGGG-3′), miR-877-5p inhibitor (5′-CCCUGCGCCAUCUCCUCUAC-3′), small interfering RNA (siRNA) targeting EIF4G2 (si-EIF4G2, sense, 5′-GCUUCUCGUUUCAGUGCUUTT-3′, antisense, 5′-AAGCACUGAAACGAGAAGCTT-3′), pcDNA 3.1 vector containing EIF4G2 (oe-EIF4G2), and their corresponding negative controls (GenePharma, Shanghai, China) were transfected into MC3T3-E1 cells at a concentration of 10 nM using Lipofectamine RNAiMAX transfection reagent (Invitrogen). After transfection, osteogenic induction was performed.

### RNA quantification

Total RNA was extracted from cells using TRIzol reagent (Thermo fisher, USA). The purity and concentration of RNA were spectrophotometrically analyzed using a Nanodrop One (Thermo Fisher). To determine miRNA, cDNA was transcribed using the TaqMan MicroRNA Reverse Transcription Kit (Thermo Fisher). To determine mRNAs, cDNA was transcribed using the First Strand cDNA Synthesis Kit (Beyotime, China). PCR was performed on an ABI 7500 Real-Time PCR system (Thermo Fisher) using the TB Green Premix Ex Taq II (Taraka, Japan). U6 was the reference gene for miRNA in the cell, and glyceraldehyde-3-phosphate dehydrogenase (GAPDH) was the reference gene for mRNA in the cell. The gene expressions were quantified using the 2^−ΔΔCT^ method. Sangong (Shanghai, China) was commissioned to synthesize primers (Table 1).

**Table 1:** PCR primers

Genes	Primers (5′–3′)
miR-877-5p	Forward: GCGCGUAGAGGAGAUGGC
	Reverse: CAGTGCGTGTCGTGGAGT
EIF4G2	Forward: CCGTTGTGACTGACTATC
	Reverse: GCTATCCCTTCCTGTTTG
ALP	Forward: CCCAGACACAAGCATTCC
	Reverse: TCAATCCTGCCTCCTTCC
COLIA1	Forward: GACGCCATCAAGGTCTAC
	Reverse: CTCGAACGGGAATCCATC
OPN	Forward: CACTCCAATCGTCCCTACAG
	Reverse: CCTTAGACTCACCGCTCTTC
β-catenin	Forward: AGACAGCTCGTTGTACTGCT
	Reverse: GTGTCGTGATGGCGTAGAAC
RUNX2	Forward: GTCCTATGACCAGTCTTA CC
	Reverse: GATGAAATGCCTGGGAACTG
U6	Forward: CTCGCTTCGGCAGCACA
	Reverse: AACGCTTCACGAATTTGCGT
GAPDH	Forward: CATCAACGGGAAGCCCATC
	Reverse: CTCGTGGTTCACACCCATC

miR-877-5p, microRNA-877-5p; EIF4G2, Eukaryotic Translation Initiation Factor 4γ2; ALP, alkaline phosphatase; COLIA1, collagen type I a1 chain; OPN, osteopontin; GAPDH, glyceraldehyde-3-phosphate dehydrogenase

### Western blot

Total proteins were extracted using radioimmunoprecipitation assay buffer (pH 7.4) and separated on 10% sodium dodecyl sulfate–polyacrylamide gel electrophoresis. Proteins transferred to nitrocellulose membranes (Millipore) were mixed with primary antibodies EIF4G2 (1:1000, ab97302, Abcam) and GAPDH (1:1000, ab8245, Abcam) after being blocked with 5% low-fat milk. Next, horseradish peroxidase-conjugated secondary antibody (1:5000, ab205718, Abcam) was supplemented to develop protein bands using enhanced chemiluminescence reagents (Amersham Biosciences, Piscataway, NJ, USA).

### ALP staining and alizarin red staining

For ALP staining, after induction for 14 days, cells were fixed with 95% ethanol and stained with BCIP/NBT solution according to the manufacturer's protocol (Beyotime Institute of Biotechnology) at room temperature for 2 h. The ALP-positive cells were stained blue/purple. Stained cells were visualized using the Canon IXUS210 camera (Canon, Inc.; magnification). In addition, an Alkaline Phosphatase Staining Kit (BestBio, Inc.) was used to detect the ALP activity, according to the manufacturer's protocol.

For alizarin red staining, after induction for 14 days, cells were washed one or two times with phosphate-buffered saline (PBS), fixed with 95% ethanol for 10 min, washed one or two times with PBS again, covered, and stained with 0.1% alizarin red solution for 10 min. Finally, they were rinsed with PBS and observed under an inverted light microscope. For quantification analysis, 10% hexadecyl pyridinium chloride monohydrate (CPC; Sigma-Aldrich; Merck KGaA) was used to dissolve the mineralized nodules and then the absorbance was measured at 540 nm using a Multiskan™ FC spectrophotometer (Thermo Fisher Scientific, Inc.).

### Dual luciferase activity assay

The amplified EIF4G2 fragment containing the miR-877-5p binding site was cloned into the psi-CHEK2 vector (Promega) to obtain the EIF4G2 wild-type (WT). Also, an EIF4G2 mutant (MUT) vector was produced. MC3T3-E1 cells co-transfected with the above plasmids and miR-877-5p mimic or NC using Lipofectamine 2000 (Invitrogen). Luciferase activity was determined using a dual luciferase reporter assay system (Promega).

### Statistical analysis

Statistical analysis was performed using PRISM v5.0 software (GraphPad, La Jolla, CA, USA). All data in three replicates are expressed as mean standard deviation and assessed by Wilcoxon test. *P* < 0.05 was considered statistically significant.

## Results

### High miR-877-5p and low EIF4G2 in osteoblast differentiation

**Fig. 1:**
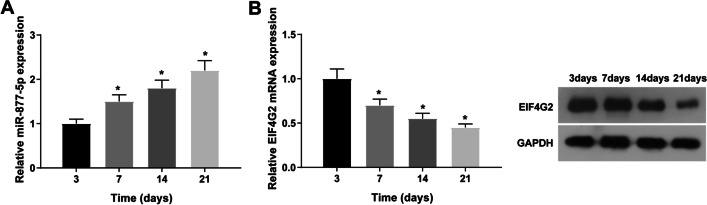
High miR-877-5p and low EIF4G2 during osteoblast differentiation. A/B: RT-qPCR or Western blot to determine miR-877-5p and EIF4G2 in MC3T3-E1 cells on days 3, 7, 14, and 21 of osteoblast differentiation; values are expressed as mean ± standard deviation; *P < 0.05 versus day 3

At 3, 7, 14, and 21 d of osteoblast differentiation, miR-877-5p and EIF4G2 levels were quantified: miR-877-5p expression increased over time in MC3T3-E1 cells, whereas EIF4G2 expression decreased (Fig. 1A, B).

### Promoting effects of miR-877-5p on osteoblast differentiation

**Fig. 2:**
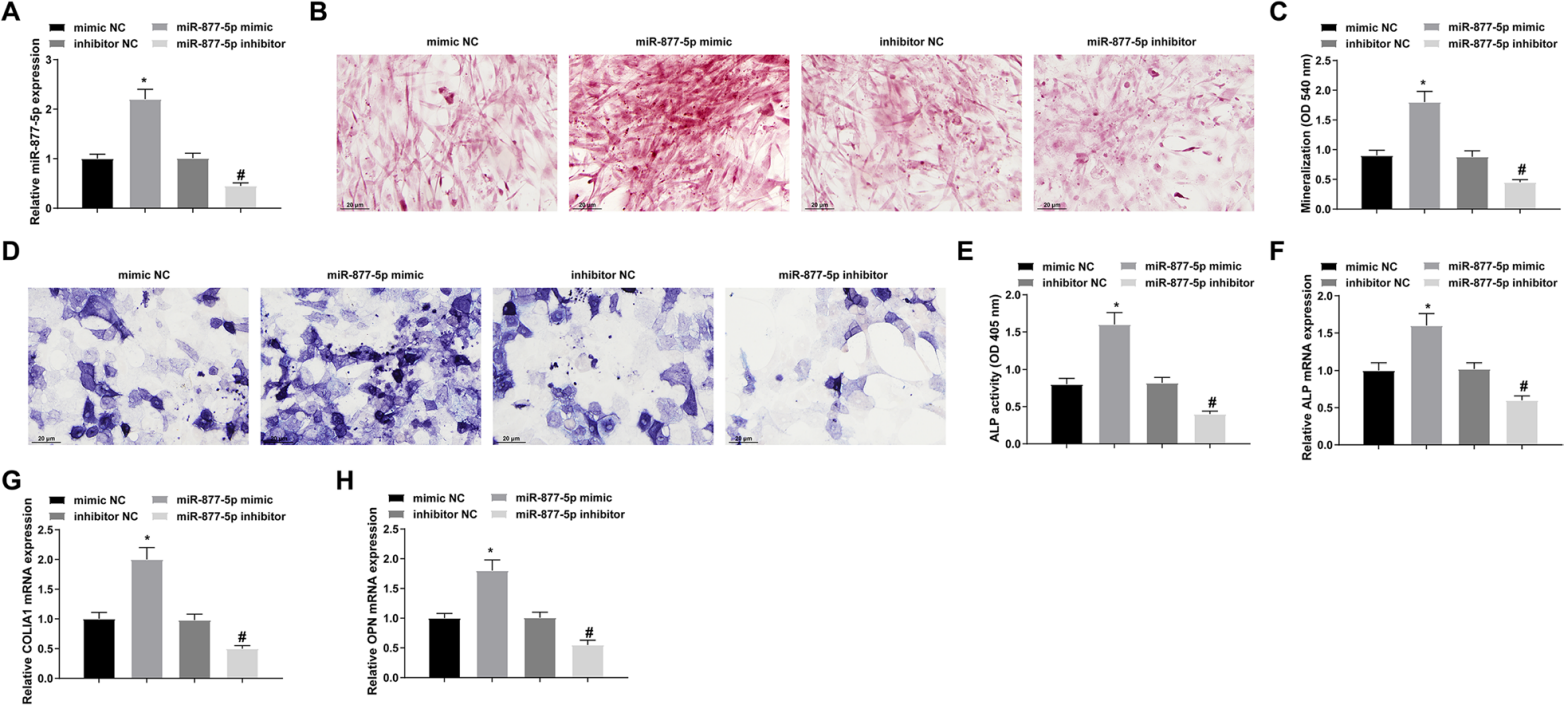
Promoting effects of miR-877-5p on osteoblast differentiation. (**A**) RT-qPCR to verify the successful transfection of miR-877-5p mimic or inhibitor; (**B**/**C**) alizarin red staining to detect the mineralization of MC3T3-E1 cells; (**D**) ALP activity assay; (**E**–**G**) RT-qPCR detection of ALP, COLIA1, and OPN; values are expressed as mean ± standard deviation; *P < 0.05 versus mimic NC; #*P* < 0.05 versus inhibitor NC

Transfection with miR-877-5p mimic or miR-877-5p inhibitor and their negative control (NC) was implemented before osteoblast differentiation. RT-qPCR results confirmed that the vectors were successfully transfected (Fig. 2A). After 14 days of osteogenic induction, alizarin red staining showed that mineralization of MC3T3-E1 cells increased after miR-877-5p upregulation (Fig. 2B, C). After 14 days of osteogenic induction, ALP staining was performed. The results showed that the activity of ALP increased after upregulation of miR-877-5p (Fig. 2D, E). In addition, miR-877-5p upregulation elevated mRNA expression of osteoblast markers ALP, COLIA1, OPN, β-catenin, and RUNX2 (Fig. 2F–H, Additional file 1: Fig. S1A). In contrast to that, miR-877-5p downregulation caused the negative results (Fig. 2B–H, Supplementary Fig. 1A).

### A negative relation between miR-877-5p and EIF4G2

**Fig. 3:**
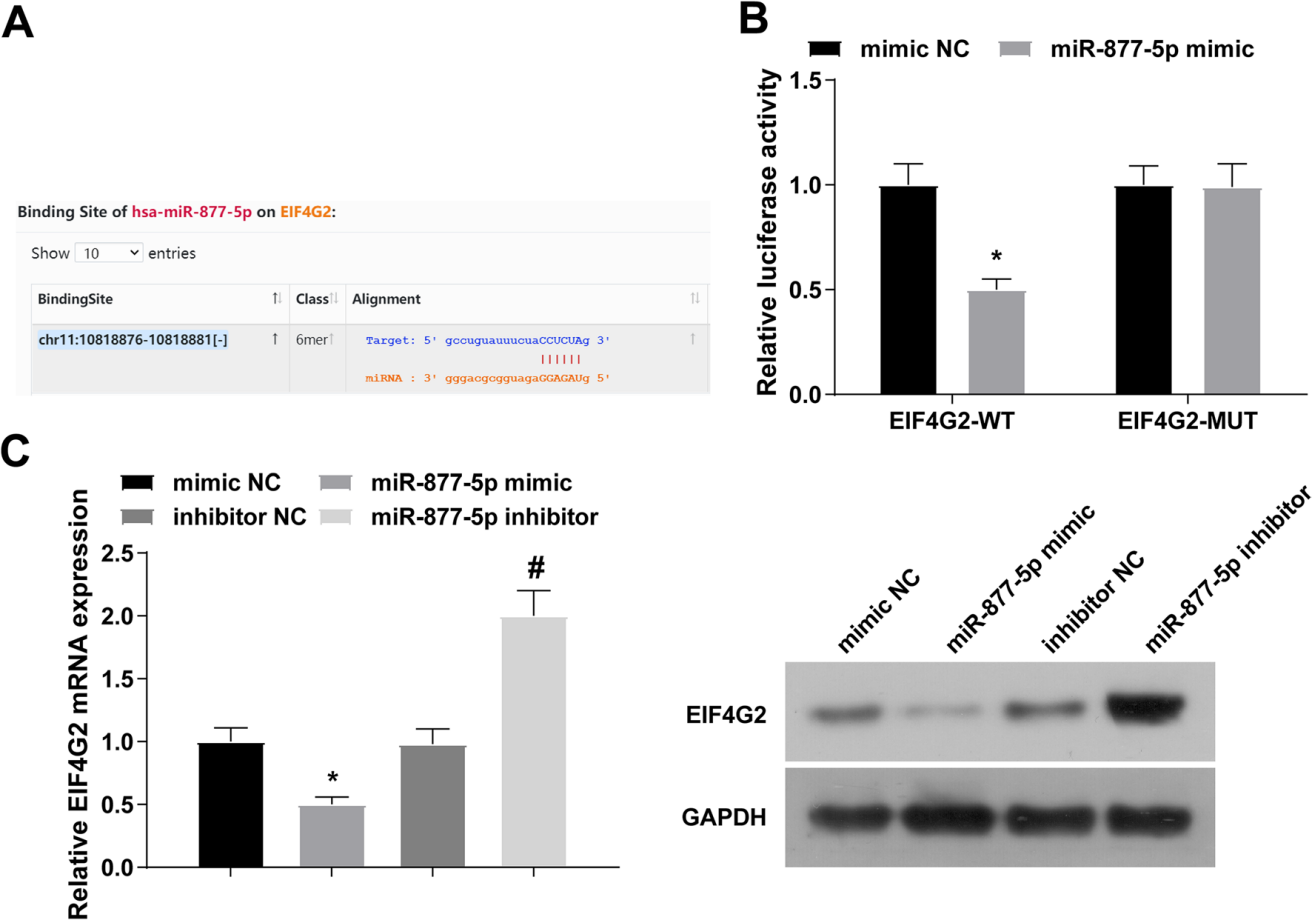
A negative relation between miR-877-5p and EIF4G2. A: starBase predicted the binding sites of miR-877-5p and EIF4G2; B: dual luciferase reporter gene detection confirmed that miR-877-5p targeted binding to EIF4G2; C: RT-qPCR and Western blot detection of EIF4G2; the values are expressed as mean ± standard deviation; *P < 0.05 versus mimic NC; #*P* < 0.05 versus inhibitor NC

Potential targets of miR-877-5p were predicted from three different algorithms: starBase (http://starbase.sysu.edu.cn/), PITA (http://genie.weizmann.ac.il/pubs/mir07/mir07_data.html), and miRanda (http://www.microrna.org/microrna/home.do). A putative miR-877-5p binding site was found in the EIF4G2 3’UTR (Fig. 3A). Luciferase activity was reduced in MC3T3-E1 cells co-transfected with miR-877-5p mimic and EIF4G2-WT (Fig. 3B). In addition, EIF4G2 expression was suppressed after miR-877-5p mimic transfection in MC3T3-E1 cells, while those transfected with miR-877-5p inhibitor had elevated EIF4G2 expression (Fig. 3C).

### Suppressive effects of EIF4G2 on osteoblast differentiation

**Fig. 4:**
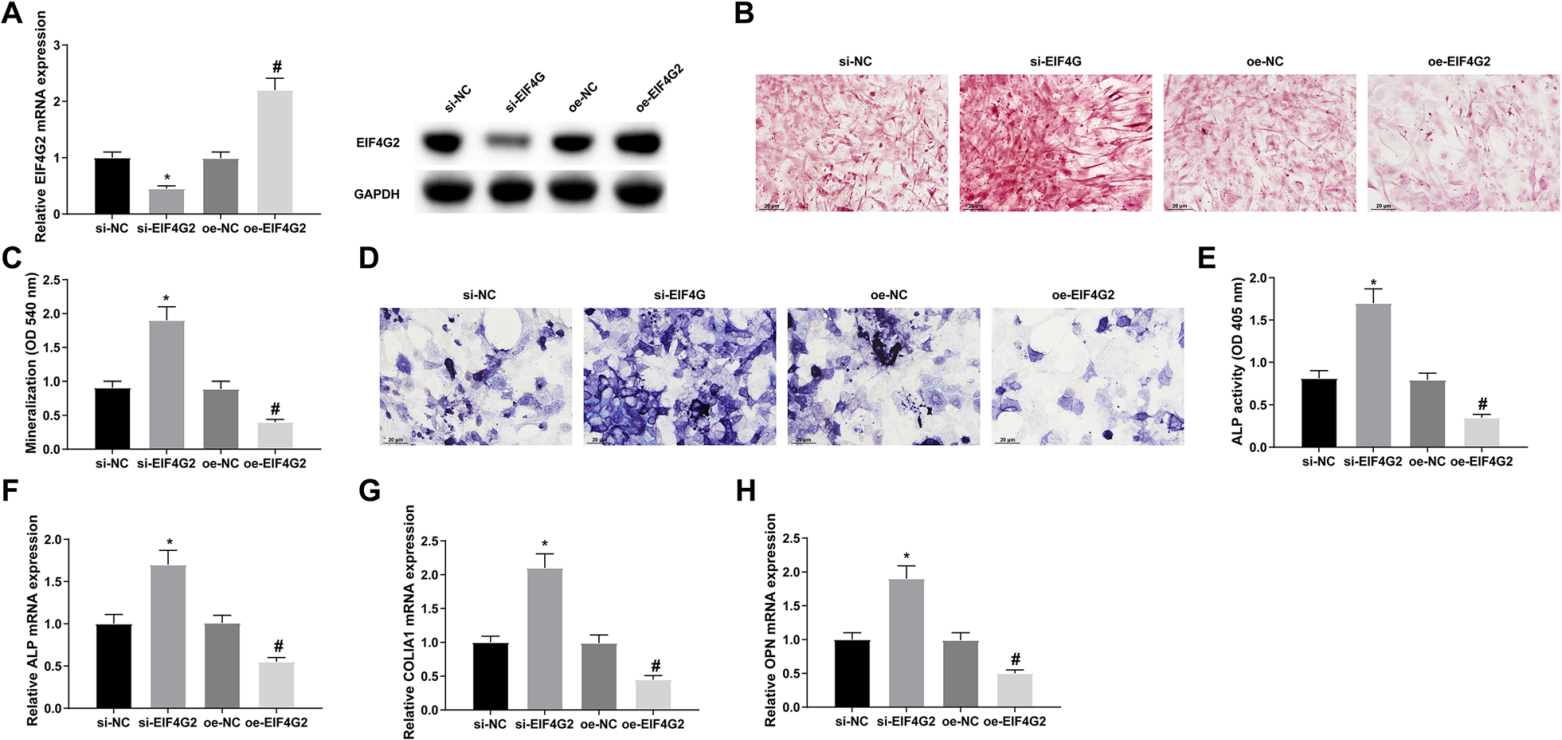
Suppressive effects of EIF4G2 on osteoblast differentiation. (**A**) RT-qPCR and Western blot to verify the successful transfection of si-EIF4G2 or oe-EIF4G2; (**B**/**C**) alizarin red staining to detect the mineralization of MC3T3-E1 cells; (**D**) ALP activity assay; (**E**–**G**) RT-qPCR detection of ALP, COLIA1, and OPN; the values are expressed as mean ± standard deviation; *P < 0.05 versus si-NC; #*P* < 0.05 versus oe-NC

Transfection with si-EIF4G2 or oe-EIF4G2 was performed before osteoblast differentiation induction, and the vectors were successfully transfected (Fig. 4A). Responded to EIF4G2 knockdown, increased mineralization, ALP activity, and expression of osteoblast markers were seen in MC3T3-E1 cells; EIF4G2 overexpression had the opposite effect (Fig. 4B-H, Additional file 1: Fig. S1B).

### Overexpression of EIF4G2 reverses the promotion of miR-877-5p upregulation on osteoblast differentiation

**Fig. 5:**
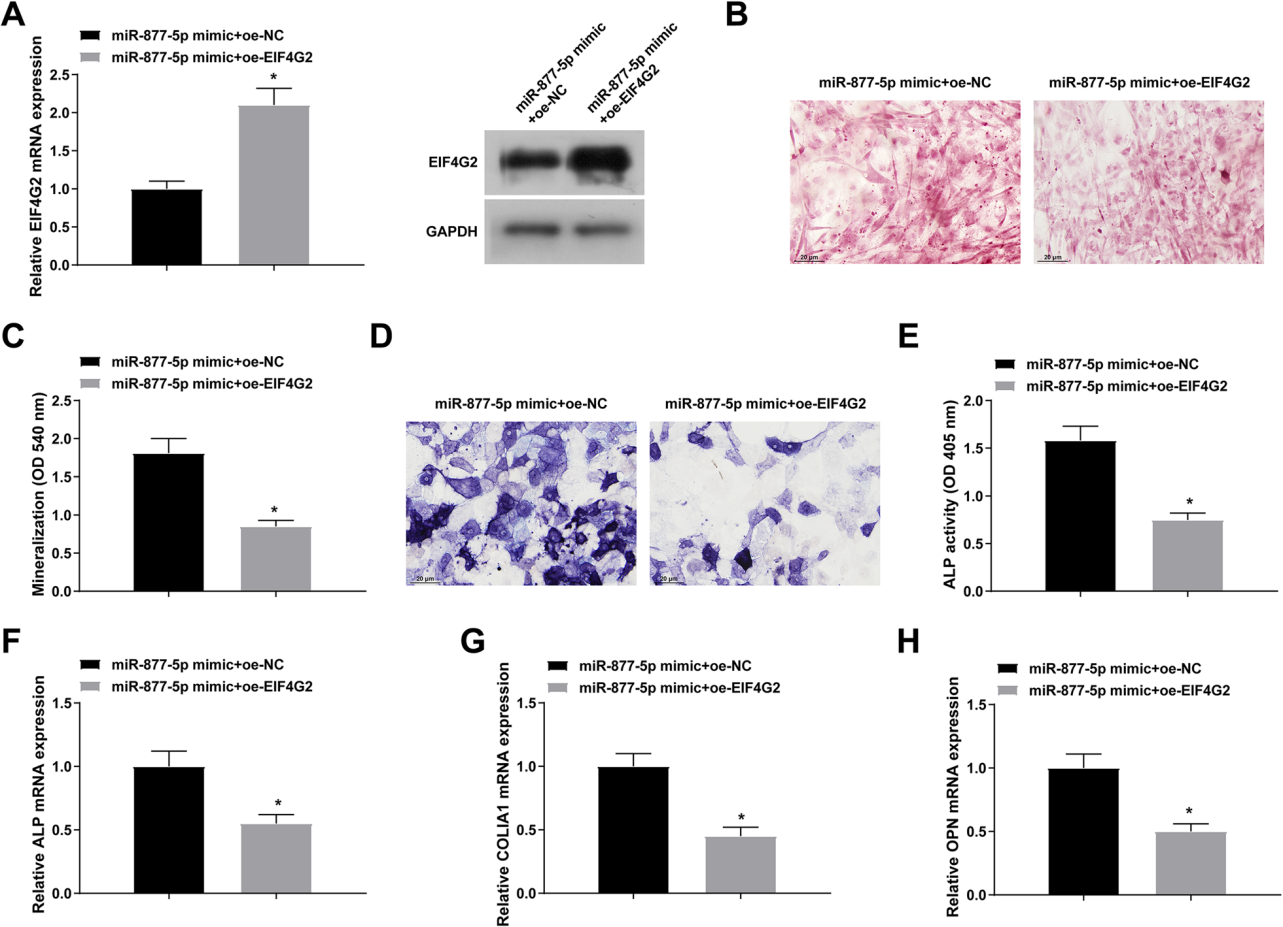
EIF4G2 reverses the promotion of miR-877-5p upregulation on osteoblast differentiation. (**A**) RT-qPCR and Western blot to verify the successful co-transfection of miR-877-5p mimic and oe-EIF4G2; (**B**/**C**) alizarin red staining to detect the mineralization of MC3T3-E1 cells; D: ALP activity assay; (**E**–**G**) RT-qPCR detection of ALP, COLIA1, and OPN; the values are expressed as mean ± standard deviation; *P < 0.05 versus miR-877-5p mimic + oe-NC

To investigate the relationship between miR-877-5p and EIF4G2 in more detail, co-transfection with miR-877-5p mimic and oe-EIF4G2 was carried out in MC3T3-E1 cells. oe-EIF4G2 not only abolished the suppressive impact of miR-877-5p mimic on EIF4G2 expression (Fig. 5A), but the promoting effect on osteoblast differentiation (Fig. 5B-G, Additional file 1: Fig. S1C).

## Discussion

Osteoblast differentiation is an essential component of bone remodeling to maintain bone health [26]. Inappropriate regulation of bone formation is associated with diseases such as osteoporosis, osteoarthritis, and bone cancer. The osteoblast differentiation process involves the regulation of gene expression, osteoblast differentiation marker genes, and mineral deposition [7–9]. Currently, many miRNAs emphasize great roles in skeletal development and homeostasis, especially in osteoblast differentiation [27–29]. Therefore, our study investigated miR-877-5p in osteoblast differentiation from the perspective of gene regulation and concluded that miR-877-5p promotes osteoblast differentiation by negatively regulating EIF4G2 expression. This is the first demonstration of the role and potential mechanism of miR-877-5p in osteogenesis.

MC3T3-E1 osteoblast cell line can be used as a host for in vitro studies of bone remodeling and formation [30–33] due to its pre-osteoblast phenotype, and its subclone 14 can mineralize the collagen extracellular matrix [34]. Therefore, mouse osteogenic MC3T3-E1 cells were applied to induce osteoblast differentiation model. Numerous studies have shown that miRNAs could act as key modulators in osteoblastic differentiation. MiR-497-5p stimulates osteoblast differentiation through HMGA2-mediated JNK pathway [30]. MiR-135-5p promotes osteoblast differentiation by targeting HIF1AN in MC3T3-E1 cells [35]. In addition, miR-532-3p inhibits osteogenic differentiation in MC3T3-E1 cells by downregulating ETS1 [36]. In this study, it was found that miR-877-5p was upregulated during the osteogenic differentiation of MC3T3-E1. Osteogenesis-related markers in osteoblast differentiation, including OPN, COLIA1, and ALP, are at high levels during extracellular matrix maturation and mineralization [37, 38]. Furthermore, mineralization of the extracellular matrix represents the end stage of osteoblast differentiation and is considered a sign of osteoblast maturation [39, 40]. Here, we found that miR-877-5p upregulation increased the mineralization of MC3T3-E1 cells and induced ALP activity and osteoblast markers’ expression.

miRNAs reduce the translation and/or degradation of target gene expression by targeting the 3′-UTR of mRNA [41]. To explore the molecular mechanism of miR-877-5p regulating osteogenic differentiation of MC3T3-E1 cells, potential targets of miR-877-5p were predicted from three different algorithms: starBase (http://starbase.sysu.edu.cn/), PITA (http://genie.weizmann.ac.il/pubs/mir07/mir07_data.html), and miRanda (http://www.microrna.org/microrna/home.do). EIF4G2, a downregulated actor during osteogenic differentiation, was validated as a downstream gene of miR-877-5p. EIF4G2 plays an important role during cell mitosis [42] and can be involved in different types of cell differentiation, such as myeloid monocytes [43], myogenic cells [44], and embryonic stem cells [45]. Recently, Hu et al. found that EIF4G2 has regulatory effects on chondrocyte viability, colony formation, and migration [46]. For novelty, our results demonstrated that downregulation of EIF4G2 promoted osteoblast differentiation, whereas upregulation of EIF4G2 inhibited osteoblast differentiation, and upregulation of EIF4G2 reversed the promoting effect of upregulation of miR-877-5p on osteoblast differentiation.

Nonetheless, the results are only applicable to in vitro osteoblast models and it is unclear whether miR-877-5p and EIF4G2 have similar effects in animal models. Since bone formation can be mediated by the recruitment of mesenchymal stem cells [47], the process of MSC differentiation into osteoblasts should be further explored.

## Conclusion

We reveal for the novelty that miR-877-5p promotes osteoblast differentiation by targeting and negatively regulating EIF4G2 expression. Therefore, therapeutic strategies targeting miR-877-5p may promote bone formation and may be effective in treating orthopedic diseases.

### Supplementary Information

**Additional file 1: Supplementary Fig. S1**. Regulatory effects of miR-877-5p/EIF4G2 axis on β-catenin and RUNX2 expression. A-C: RT-qPCR to detect β-catenin and RUNX2; the values are expressed as mean ± standard deviation; *P < 0.05 versus mimic NC; #*P* < 0.05 versus inhibitor NC; + *P* < 0.05 versus si-NC; ^*P* < 0.05 versus oe-NC; & *P* < 0.05 versus miR-877-5p mimic + oe-NC.

## Data Availability

Data are available from the corresponding author on request.
