# Supplementary figures and images for: MicroRNA-877-5p promotes osteoblast differentiation by targeting EIF4G2 expression
Source: J Orthop Surg Res. 2024 Feb 12;19:134. doi: 10.1186/s13018-023-04396-y (PMC10860299; doi:10.1186/s13018-023-04396-y)

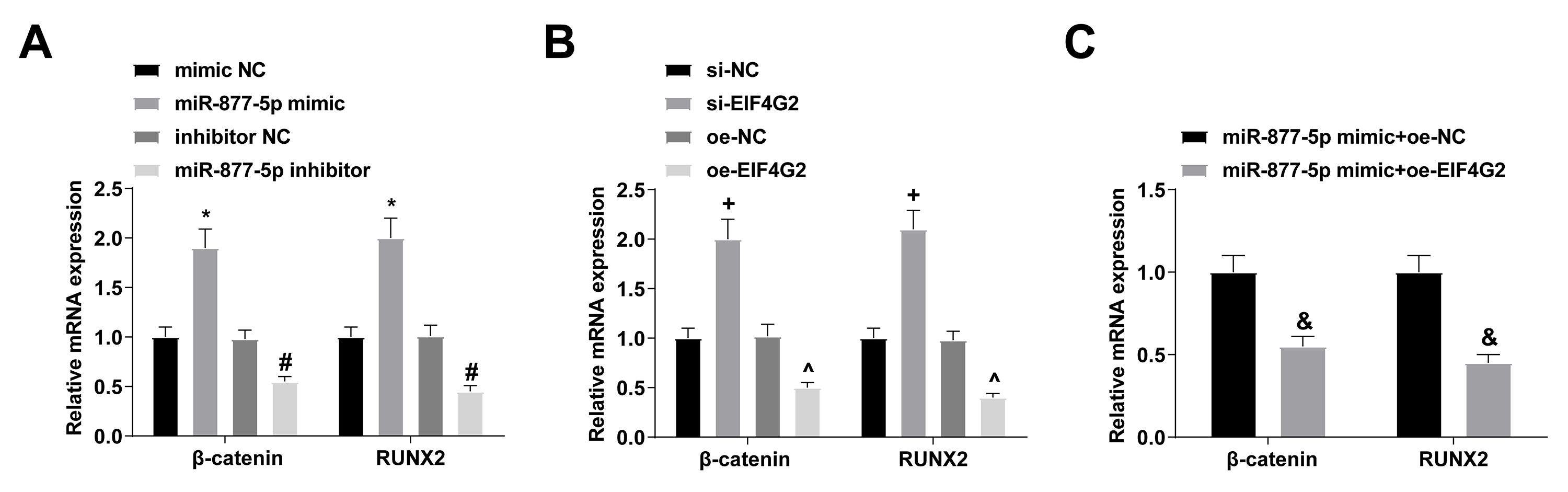

Supplement: Supplementary file 1 — Additional file 1: Supplementary Fig. S1. Regulatory effects of miR-877-5p/EIF4G2 axis on β-catenin and RUNX2 expression. A-C: RT-qPCR to detect β-catenin and RUNX2; the values are expressed as mean ± standard deviation; *P < 0.05 versus mimic NC; #P < 0.05 versus inhibitor NC; + P < 0.05 versus si-NC; ^P < 0.05 versus oe-NC; & P < 0.05 versus miR-877-5p mimic + oe-NC. [file 13018_2023_4396_MOESM1_ESM.tif]
